# Effects of Keratinocyte-Derived Cytokine (CXCL-1) on the Development of Theiler's Virus-Induced Demyelinating Disease

**DOI:** 10.3389/fcimb.2018.00009

**Published:** 2018-01-23

**Authors:** Min H. Kang, Young H. Jin, Byung S. Kim

**Affiliations:** Department of Microbiology-Immunology, Northwestern University Medical School, Chicago, IL, United States

**Keywords:** CXCL-1 chemokine, keratinocyte-derived cytokine, infection and replication, central nervous system, recombinant TMEV

## Abstract

CXCL-1, also called keratinocyte-derived cytokine (KC), is a predominant chemokine produced in glial cells upon infection with Theiler's murine encephalomyelitis virus (TMEV). In this study, we assessed the role of KC in the development of TMEV-induced demyelinating disease by utilizing polyclonal anti-KC antibodies as well as KC-expressing recombinant TMEV. Our results indicate that the level of KC produced after infection with TMEV or stimulation with various TLRs is significantly higher in various cells from susceptible SJL mice compared to those in cells from resistant B6 mice. SJL mice treated with rabbit anti-KC antibodies displayed accelerated development of TMEV-induced demyelinating disease, elevated viral loads in the CNS and decreased antiviral T cell responses. In addition, infection of susceptible SJL mice with recombinant KC-TMEV produced biologically active KC, which resulted in the accelerated pathogenesis of demyelinating disease and elevated T cell responses to viral antigens compared to mice infected with control recombinant HEL-TMEV. These results strongly suggest that both the lack of KC during TMEV infection and the excessive presence of the chemokine promote the pathogenesis of demyelinating disease. Therefore, a balance in the level of KC during TMEV infection appears to be critically important in controlling the pathogenesis of demyelinating disease.

## Introduction

Theiler's murine encephalomyelitis virus (TMEV)-induced demyelinating disease in mice has been extensively investigated as a relevant animal model for multiple sclerosis (MS). Infection of susceptible SJL/J mice with TMEV causes the development of clinical signs similar to MS (Leibowitz and Rodriguez, 1983; Dal Canto et al., 1996; Kim et al., 2001). Immune responses to viral and/or CNS autoantigens have been implicated in the development of TMEV-induced demyelinating disease (Lipton and Dal Canto, 1976; Clatch et al., 1987; Yauch et al., 1998). TMEV has a single-stranded RNA genome of ~8 kb with positive polarity and belongs to the genus *Cardiovirus* of the *Picornaviridae* family (Lehrich et al., 1976; Lorch et al., 1981). TMEV infects various glial cells, antigen-presenting macrophages and dendritic cells (Aubert et al., 1987; Zheng et al., 2001; Lipton et al., 2005; Jin et al., 2007; Hou et al., 2009). TMEV infection activates the production of various proinflammatory cytokines and chemokines in these cell types (Palma and Kim, 2001, 2004). These proinflammatory chemokines and cytokines consequently contribute to cellular infiltration into the CNS and further activation of infiltrated inflammatory immune cells, eventually leading to the development and progression of virus-induced demyelinating disease (Kim et al., 2001, 2012; Hou et al., 2009).

Infection of cells with TMEV activates the production of various cytokines and chemokines via TLR- and melanoma differentiation-associated gene 5 (MDA5)-dependent pathways (So et al., 2006; So and Kim, 2009; Jin et al., 2012). Although an excessive level of TLR3 signaling contributes to the pathogenesis of the disease, the presence of the TLR3 signal is required to protect the host from chronic viral infections (Jin et al., 2011). These results suggest that the level of activating signaling and downstream cytokine production is critically important in the protection or the pathogenesis of demyelinating disease. In fact, the presence of high levels of IL-1 or type I IFNs, which are downstream products of such activations, play a pathogenic role. However, the lack of these cytokines promotes viral persistence and manifestation of the disease (Jin et al., 2010; Kim et al., 2012). Similarly, excessive levels of IL-6, which has important roles in B cell development and T cell responses, exert a potent effect on the pathogenesis of TMEV-induced demyelinating disease by promoting Th17 responses (Hou et al., 2009). Therefore, understanding the roles of such cytokines and chemokines produced upon viral infections in the protection and/or pathogenesis of chronic viral diseases is of paramount importance.

Very little attention has been given to the role of chemokines produced upon viral infections in the protection and/or pathogenesis of viral diseases. The major function of chemokines is to attract various cells and promote the migration of cells to certain sites. It has been long known that TMEV infection, similar to other viral infections, induces production of various chemokines in different types of glial cells and antigen-presenting cells such as DCs and macrophages (Palma and Kim, 2001, 2004). KC (keratinocyte-derived cytokine), an innate chemokine encoded by a short gene (<250 bp), is the predominant chemokine produced by various glial cell types after infection with TMEV (Palma and Kim, 2004; Rubio and Sanz-Rodriguez, 2007). The level of KC secreted by astrocytes 6 h after TMEV infection was more than 20-fold higher compared to that of MCP-1 and MIP-1α (Palma and Kim, 2004). KC is also known as GRO1 oncogene, GROα, or CXCL-1. It is known to be associated with infiltration of neutrophils and other inflammatory cells (Zhou et al., 2003) that are believed to contribute to the pathogenesis of TMEV-induced demyelinating disease in the CNS (Aubert et al., 1987; Zheng et al., 2001; Jin et al., 2007, 2015). It has also previously shown that the lack of KC and sustained presence of KC amplifies the development of mouse hepatitis virus-induced demyelinating disease (Hosking et al., 2009; Marro et al., 2016). In addition, KC signaling via CXCR2 is also associated with the number and positioning of oligodendrocytes during the development of the spinal cord (Robinson et al., 1998; Tsai et al., 2002). Furthermore, KC signaling appears to associate with neuroprotection (Omari et al., 2009) or pathogenesis in different MS models (Kerstetter et al., 2009). However, the underlying mechanisms for the effects of KC associated with viral infection on the development of TMEV-induced demyelinating disease remain unclear.

In this study, we assessed the role of KC in the development of TMEV-induced demyelinating disease by utilizing polyclonal anti-KC antibodies and KC-expressing recombinant TMEV (KC-TMEV). Our results indicate that the level of KC produced after infection with TMEV or stimulation with various TLRs is significantly higher in cells from susceptible SJL mice compared to those in cells from resistant B6 mice, suggesting an important role of KC in the susceptible mice. SJL mice treated with rabbit anti-KC antibodies displayed accelerated development of TMEV-induced demyelinating disease, reduced cellular infiltration to the CNS and decreased levels of antiviral T cell responses, accompanied with increased viral loads in the CNS. In addition, we generated a stable recombinant KC-expressing TMEV strain to investigate the role of chemokine production at the site of TMEV infection during the pathogenesis of demyelinating disease. KC produced in the non-mouse cells (BHK) after infection with the recombinant KC-TMEV yielded a biologically active chemokine. The levels of chemokine produced in mouse cells infected with TMEV were significantly higher than that in cells infected with control HEL-TMEV infection. In addition, infection of susceptible SJL mice with KC-TMEV resulted in accelerated pathogenesis of demyelinating disease along with elevated levels of both pathogenic and protective T cell responses to viral antigens compared to mice infected with control HEL-TMEV. These results suggest that both the lack of KC during TMEV infection and the excessive presence at the site of infection promote the pathogenesis of demyelinating disease. Therefore, a balance in the KC level appears to be critically important in controlling the pathogenesis of TMEV-induced demyelinating disease.

## Methods

### Animals

Female SJL/J and C57BL/6 mice (4- to 6-weeks-old) were purchased from Harlan (Indianapolis, IN). The mice were housed in the animal facility of the Northwestern University Medical School and used following protocols approved by the Institutional Animal Care and Use Committee.

### Preparation of macrophages and astrocytes

Peripheral macrophages were obtained from the adherent population of peritoneal exudates of adult mice. The majority (>90%) of the adherent cells were CD45^+^CD11b^+^. Primary astrocytes were derived from 0- to 3-day-old neonates by using differential shaking (Skias et al., 1987). The cell preparations were at least 90% pure, as confirmed by staining with antibodies specific for the astrocyte marker GFAP (Dako, California).

### *In vitro* stimulation and viral infection

For *in vitro* viral infection, cells were washed and subsequently incubated in infection media (DMEM supplemented with 0.1% bovine serum albumin) with the BeAn strain of TMEV at various multiplicity of infection (MOI). Macrophages and astrocytes derived from susceptible SJL and resistant B6 mice (5 × 10^5^/well) in six-well plates were infected with TMEV (at MOI = 1, 5, or 10) in triplicates for 24 h. In addition, macrophages from MyD88-deficient (009088/MyD88^tm1.1Defr^, Jackson laboratory) or control B6 mice were similarly stimulated with TMEV infection (MOI = 10 or 50), or treatment with TLR ligands or TNF-α for 24 h.

### Virus preparation and assessment

The BeAn strain of TMEV was expanded in BHK cells, which were grown in Dulbecco's modified Eagle's medium (Thermo-Fisher, Waltham, MA) supplemented with 7.5% donor calf serum (Yauch et al., 1998). Viral titer was determined using a standard plaque assay on BHK monolayers (Pullen et al., 1995).

### Assessment of clinical signs

Approximately 30 μl of TMEV was injected into the right hemisphere of 5- to 7-week-old mice anesthetized with isoflurane. SJL mice were intraperitoneally injected with polyclonal rabbit anti-KC IgG antibodies (100 μg) or control IgG from unimmunized rabbits (Cellscience, Canton, MA) at −1, 2, and 6 dpi of TMEV (1 x 10^6^ PFU). For recombinant TMEV study, SJL mice were infected with 3 × 10^6^ PFU KC-TMEV or HEL-TMEV. The clinical symptoms of the disease were assessed weekly using the following grading scale: grade 0 = no clinical signs; grade 1 = mild waddling gait; grade 2 = moderate waddling gait and hind limb paresis; grade 3 = severe hind limb paralysis; grade 4 = severe hind limb paralysis and loss of righting reflex; and grade 5 = death.

### Elisa

The levels of cytokines/chemokines in the culture supernatants in 96-well plates after TMEV infection or mock infection with BHK lysates were assessed using specific ELISA kits. Mouse KC (CXCL-1), mouse IFN-γ, and IL-17 kits were purchased from BD Biosciences (San Diego, CA). The cytokine levels in splenic culture supernatants stimulated with viral epitopes for 72 h were assessed according to the manufacturer's instructions. Briefly, diluted samples were incubated for 2 h with the plate-bound capture antibodies after blocking for 1 h. The expression levels of the cytokines were visualized using HRP-conjugated detection antibodies in the presence of the HRP substrate tetramethylbenzidine (TMB; BioFX Laboratories, Owings Mills, MD, USA). The plates were read at 450 nm using a microplate reader.

### Histopathological examinations

At 35 days post-infection (dpi), SJL mice treated with anti-KC antibody or control antibody were perfused via intracardiac puncture with 50 ml of cold PBS. Brains and spinal cords were fixed in 4% formalin in PBS for 4 days and then embedded in paraffin for sectioning and staining. Paraffin-processed brains and spinal cords were sectioned at 6 μm thickness. Sections from each animal were deparaffinized, rehydrated, and evaluated separately by Luxol Fast Blue (LFB) staining for axonal demyelination and by hematoxylin and eosin (H&E) staining for inflammatory infiltrates. The slides were examined using Leica DMR light microscope and images were captured using AxioCam MRc camera and AxioVision imaging software.

### Preparation of CNS mononuclear cells

Brains and spinal cords were removed from mice after perfusion with Hank's balanced salt solution (HBSS) through the left ventricle. The tissues were forced through a steel mesh to prepare single-cell suspensions and incubated at 37°C for 45 min in PBS containing 250 μg/ml of collagenase type 4 (Worthington Biochemical Corp., Lakewood, NJ). A continuous 100% Percoll gradient (Pharmacia, Piscataway, NJ) was centrifuged at 27,000 g for 30 min to enrich for CNS-infiltrating mononuclear cells.

### Flow cytometry determinations

CNS mononuclear cells from mice were cultured in plates coated with either anti-CD3 plus anti-CD28 antibodies or viral epitope peptides in the presence of Golgi-Plug for 6 h at 37°C. The cells were then incubated in 50 μl of 2.4G2 hybridoma (American Type Culture Collection) supernatant for 30 min at 4°C to block the Fc receptors. The cells were incubated for an additional 30 min at 4°C in the presence of allophycocyanin-conjugated anti-CD8 (clone 53-6.7) or anti-CD4 (GK1.5) antibodies diluted in 50 μl of Fc-block (2.4G2 supernatant). After two washes, intracellular IFN-γ staining was performed according to the manufacturer's instructions (BD Pharmingen) using phycoerythrin-labeled rat monoclonal anti-IFN-γ antibody (XMG1.2). The cells were analyzed on a Becton Dickinson FACSCalibur flow cytometer.

### Generation of KC-TMEV and HEL-TMEV constructs

The Cla I/dL23 TMEV construct, which has 23 central codons of the TMEV leader replaced with the Cla I restriction site (Calenoff et al., 1995), was obtained from Dr. Calenof of Northwestern University at Northwestern University Medical School, Chicago, IL. The sense PCR primer containing the Cla I site with the 5′ mature (77 aa) KC codons (5′ATC GAT CGC CTG GCC ACA GGG GCG 3′) and anti-sense primer containing the Cla I site with the 3′ end of KC (5′ATC GAT CTT GGG GAC ACC TTT TAG 3′) were used to amplify Cla I-KC cDNA from mRNA preparations of LPS-activated macrophages. The amplified Cla I-KC cDNA was ligated into the Cla I site of dL23 TMEV. Similarly, a short form of the hen egg lysozyme (5′ ~220 bp of HEL) was amplified with Cla I-HEL primers (5′ATC GAT AAA GTC TTT GGA CGA TGT 3′ and 5′ATC GAT TGG GGT CCT GCC ATC GTT 3′) from hen embryonic library and cloned into the Cla I site of dL23 TMEV. This HEL-TMEV was used as the control for KC-TMEV.

### Western blot analysis

The uninfected control and virus-infected BHK cells were disrupted using lysis buffer (Tris-buffered saline with 1% Triton X-100). The samples (10 μγ/lane) were separated by electrophoresis on 12% sodium dodecyl sulfate (SDS) polyacrylamide gels, transferred onto nitrocellulose membrane (Amersham Pharmacia Biotech, Piscataway, NJ), and then treated with polyclonal rabbit antibodies specific for mouse KC (IBL-America, Minneapolis, MN55432). Horseradish peroxidase (HRP)-conjugated antibodies were subsequently applied as the secondary antibodies. Specific protein levels were visualized using the ECL kit (Amersham Biosciences, Piscataway, NJ).

### Measurement of neutrophil migration

Peripheral blood cells from naïve green fluorescent protein (GFP)-expressing C57BL/6 Tg mice [C57BL/6 Tg(CAG-EGFP), Jackson Laboratories, Maine] were used as the source of neutrophils. After removal of red blood cells using red blood cell lysing buffer (R7757, Sigma Aldrich), the cells were further purified using negative magnetic cell sorting, which utilizes the antibodies for CD2, CD5, CD45R, F4/80, and ICAM-1, as previously described (Cotter et al., 2001; Yona et al., 2010). A standard micropore filter assay using Boyden chambers was performed (Gho et al., 1999). Briefly, the lower well of the chamber of a 12-well plate was filled with conditioned media from BHK monolayers mock-infected and infected with TMEV, KC-TMEV, or HEL-TMEV for 24 h at 10 MOI. The upper chambers containing transwells (8 μm pore size, Corning, #353182) were placed on the conditioned media. The isolated neutrophils were added to the transwells and incubated in 5% CO_2_ for 3 h at 37°C. The number of cells on the outside of the transwell membrane were enumerated using fluorescence microscopy.

### Statistical analysis

The data are shown as the mean ± *SD* of either 2–3 independent experiments or as a representative from at least three independent experiments. The differences in the mean values were determined by Student's *t*-test using the InStat Program (GraphPad Software, San Diego, CA, USA), unless otherwise indicated. Multiple group comparisons were done by one-way analysis of variance (ANOVA) with Tukey-Kramer *post-hoc* analysis. *P*-values of <0.05 were considered to be statistically significant.

## Results

### Higher production of KC (CXCL-1) in cells derived from susceptible SJL mice compared to those from resistant B6 mice

**Figure 1:**
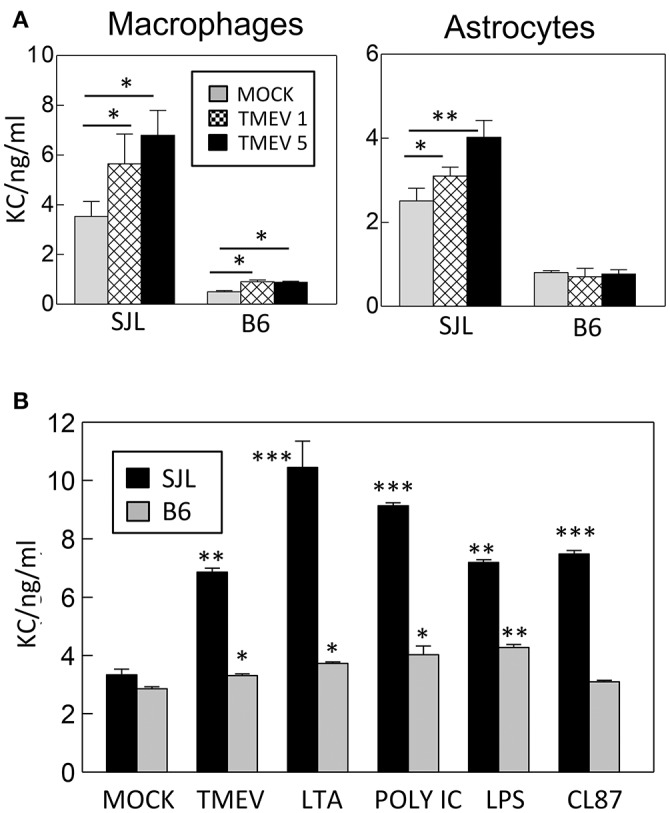
**(A)** Levels of KC (CXCL-1) produced in macrophages and astrocytes derived from susceptible SJL and resistant B6 mice after TMEV infection (MOI = 1 and 5) in triplicates for 24 h. **(B)** Levels of KC produced in peripheral macrophages from susceptible SJL and resistant B6 mice after stimulation with TMEV (MOI = 10), LTA (TLR2 ligand), poly I:C (TLR3 ligand), LPS (striTLR4 ligand), or CL87 (TLR7 ligand) for 24 h. The levels of KC protein in triplicate cultures were assessed using mouse KC specific ELISA (BD Biosciences). Multiple group comparisons were done by one-way analysis of variance (ANOVA) with Tukey-Kramer *post-hoc* analysis. Asterisks indicate the differences between the cells stimulated with TLR ligands and the corresponding control cells treated with PBS. ^*^p < 0.05; ^**^p < 0.01; ^***^p < 0.001.

To determine whether the levels of KC induction from susceptible SJL and resistant C57BL/6 mice are different after TMEV infection in cells, the levels of KC proteins produced in peripheral macrophages and primary neonatal astrocytes derived from these mice were compared using specific ELISA (Figure 1). Interestingly, the background levels of KC produced without TMEV infection were significantly high (>2-fold; *p* < 0.01) in both cell types from SJL mice compared to those from B6 mice (Figure 1A). In addition, the increase in KC production was greater in the cells after TMEV infection; consequently, the overall KC level was significantly higher (>3-fold; *p* < 0.01) in the SJL mice compared to B6 mice. Furthermore, the levels of KC production were much higher (*p* < 0.01) in macrophages from SJL mice compared to those from B6 mice after stimulation with different TLR ligands; LTA for TLR2, poly I:C for TLR3, LPS for TLR4, and CL87 for TLR7 (Figure 1B). Therefore, much higher levels of KC are produced in cells from susceptible SJL mice in response to various stimulants compared to cells from resistant B6 mice. These differences in KC production levels after TMEV infection may affect cellular migration and pathogenesis of demyelinating disease.

### MyD88-dependent activation of KC production following infection with TMEV

**Figure 2:**
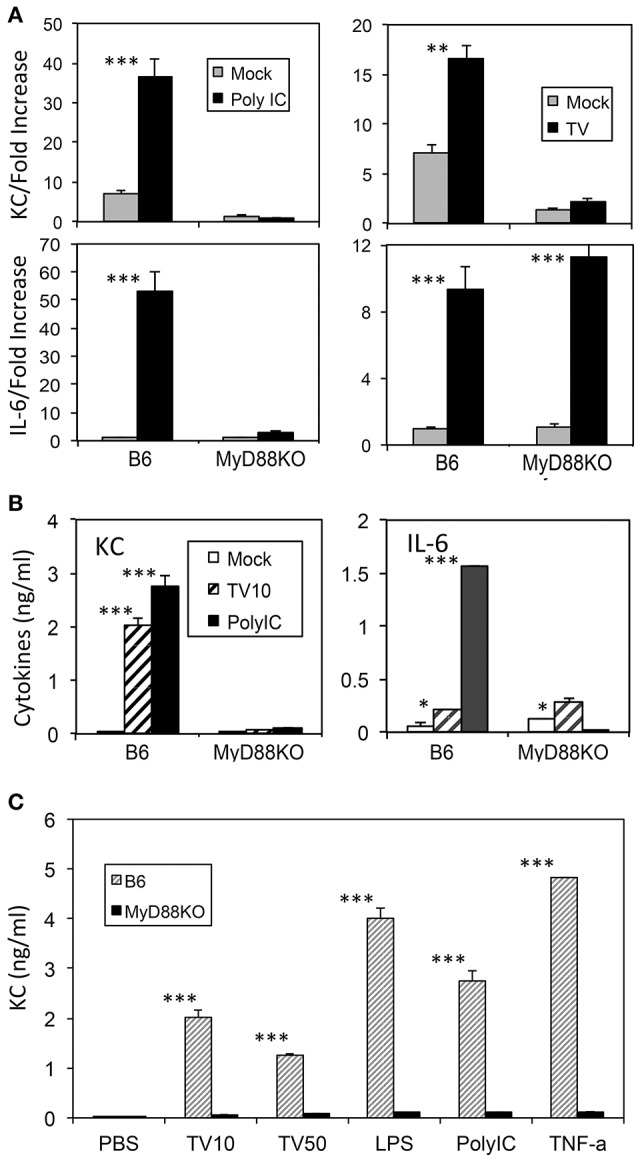
KC levels produced in macrophages with or without MyD88 signaling after stimulation with TMEV, TLR ligands, or TNF-α for 24 h **(A)** Levels of KC and IL-6 mRNAs after TMEV infection in triplicate macrophage cultures from MyD88-deficient and control B6 mice. **(B)** Levels of KC and IL-6 proteins produced in the macrophages infected with TMEV or poly I:C were assessed using ELISA. **(C)** Levels of KC produced in the macrophages with or without MyD88 after infection with TMEV (MOI = 10 or 50) or stimulation with LPS, poly I:C, or TNF-α, compared using ELISA. Multiple group comparisons were done by one-way analysis of variance (ANOVA) with Tukey-Kramer *post-hoc* analysis. ^*^p < 0.05; ^**^p < 0.01; ^***^p < 0.001.

It has previously been shown that KC production is dependent on the presence of MyD88 (Farnand et al., 2011; Guerrero et al., 2012). We examined the requirement of MyD88 in the production of KC and IL-6 after TMEV infection in macrophages derived from MyD88-deficient and control B6 mice (Figure 2). Both KC and IL-6 mRNA levels were significantly elevated (>3-fold) in macrophages from control B6 mice after treatment with a TLR3 ligand poly I:C or infection with TMEV (Figure 2A). In contrast, KC mRNA levels produced in MyD88-deficient macrophages after these stimulations were minimal, indicating that the production of KC is dependent on the presence of MyD88. Interestingly, however, IL-6 mRNA produced in MyD88-deficient macrophages was minimal after stimulation with poly I:C but was comparable to the level of control macrophages after infection with TMEV. Furthermore, KC and IL-6 protein levels assessed using ELISA were consistent with the corresponding mRNA levels in the macrophages after these stimulations (Figure 2B). This confirmed that KC production was completely dependent on the presence of MyD88 following poly I:C treatment or TMEV infection. Therefore, these results suggest that the production of KC is dependent on the presence of MyD88 but that of IL-6 is less dependent on MyD88 in the case of TMEV infection. Additionally, the dependence of KC production in the presence of MyD88 was consistent with the production after stimulation with LPS or TNF-α (Figure 2C).

### Elevated pathogenesis of TMEV-induced demyelinating disease in mice treated with anti-KC antibodies

**Figure 3:**
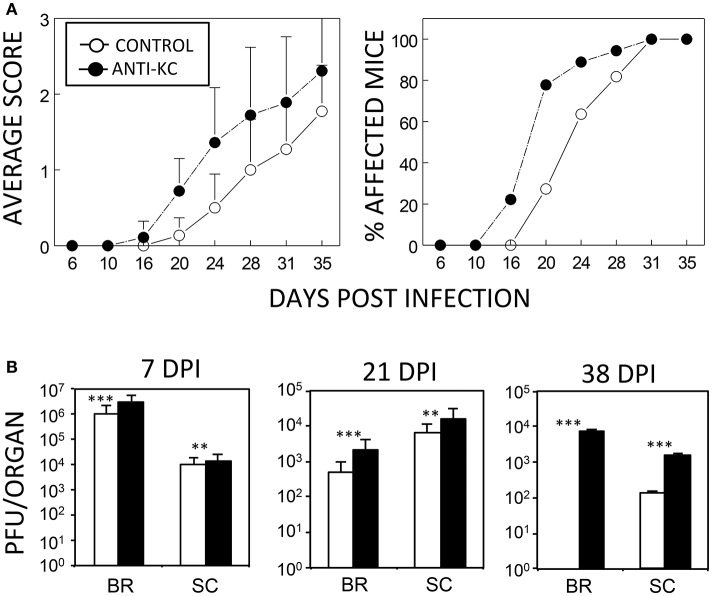
Development of TMEV-induced demyelinating disease in mice treated with anti-KC antibodies or control IgG. **(A)** Polyclonal rabbit anti-KC antibody or normal rabbit IgG (200 μg/mouse) was intraperitoneally administered into SJL mice (*n* = 18 and *n* = 11, respectively) at −1, 2, and 6 dpi with TMEV (1 × 10^6^ pfu/mouse). The infected mice were graded for clinical signs as described in the Materials and Methods section. The results were expressed as average clinical scores and percentages of the affected animals at the indicated dpi. The two-tailed *p*-value of average clinical scores between the two groups based on the paired *t*-test during days 16 and 35 post infection was very significant (*p* < 0.0027). **(B)** Levels of viral loads in the CNS of TMEV-infected anti-KC antibody-treated mice (*n* = 3) and in the control IgG-treated mice (*n* = 3) were measured using the standard plaque assay at 7, 21, and 38 dpi. ^**^p < 0.01; ^***^p < 0.001.

Although an excessive level of KC may promote the pathogenesis of the demyelinating disease, a lack of KC can also result in disease pathogenesis because of the failure to recruit cells that are required to protect against viral infection. To test this possibility, susceptible SJL mice were treated with anti-KC IgG (*n* = 18) or control IgG (*n* = 11) prior to viral infection and then the development of clinical symptoms and viral load levels were assessed (Figure 3). Treatment with anti-KC antibodies significantly promoted the pathogenesis of demyelinating disease by TMEV (*p* < 0.003) compared to mice treated with control IgG (Figure 3A). Consistently, the anti-KC antibody-treated mice also showed significantly higher levels (*p* < 0.01) of viral loads in the CNS (both brains and spinal cords) throughout the infection period (Figure 3B). Therefore, we showed that the lack of KC results in the acceleration TMEV-induced disease pathogenesis and higher viral loads in the CNS.

### Histopathological evidence of higher demyelination in SJL mice treated with anti-KC antibody compared to the control SJL mice

**Figure 4:**
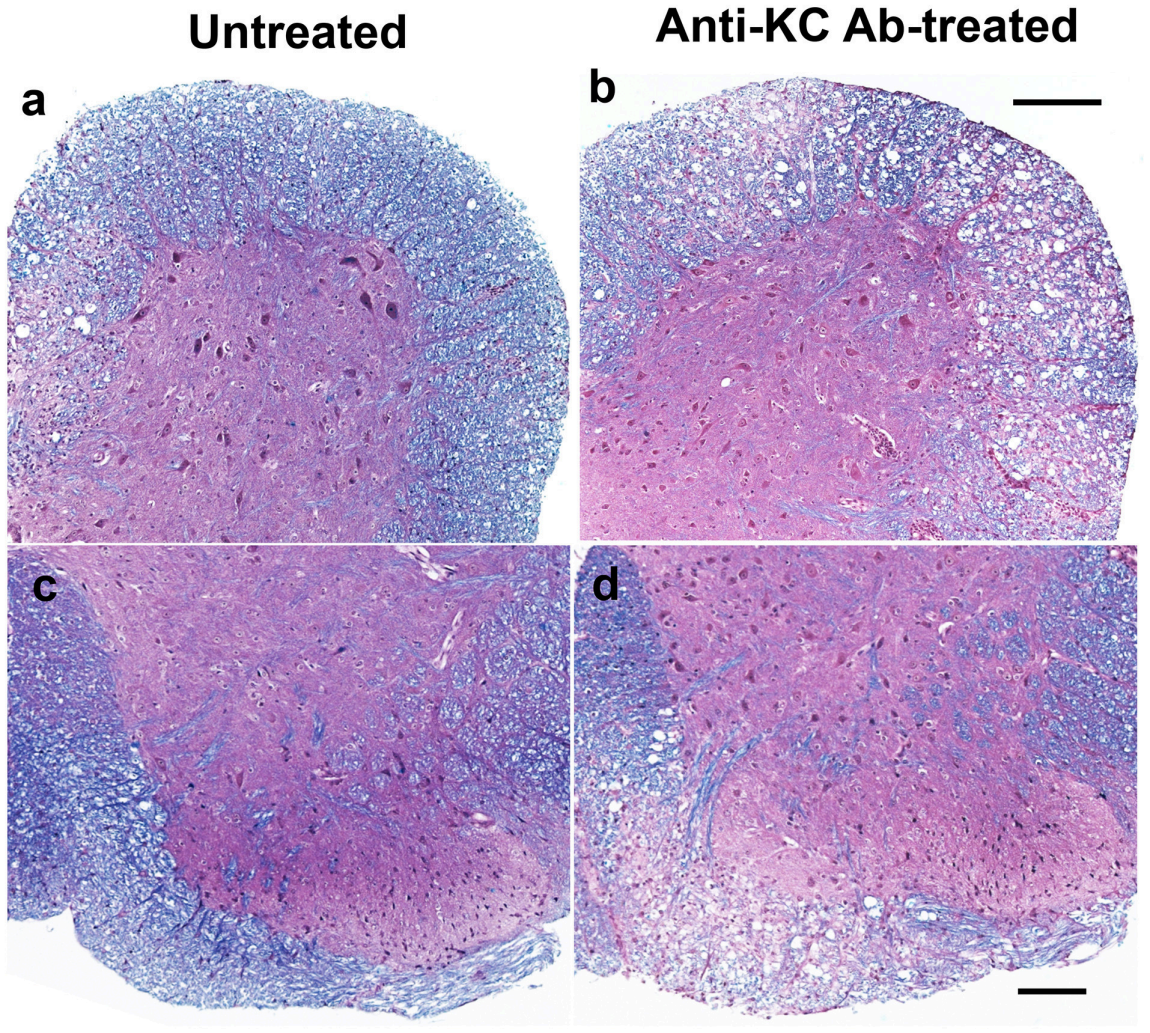
Comparison of histopathology in the spinal cords of anti-KC antibody-treated SJL mice and control SJL mice at 35 dpi. Histopathology of the spinal cords of virus-infected mice was examined using LFB and H&E staining. LFB stain showing myelin in blue in the white matter of spinal cords of anti-KC antibody-treated SJL mice **(b,d)** and SJL mice **(a,c)**; H&E stained nuclei in dark blue indicating lymphocyte infiltration in the spinal cords of anti-KC antibody-treated SJL mice **(b,d)** and SJL mice **(a,c)**; Scale bars **a**, **b** = 200 μm; **c, d** = 100 μm.

To correlate the development of clinical signs with demyelination in the CNS, histopathological examinations of the spinal cords from both anti-KC antibody-treated or control SJL mice were performed by LFB and H&E staining at 35 dpi (Figure 4). Irregular vacuolation and moderate demyelination were identified in the white matter of spinal cords in both groups of mice. Lymphocytic infiltrates were also found in the white matter of spinal cord in both groups. However, there were more severe demyelination and infiltration in spinal cords from the anti-KC antibody-treated SJL mice than in control SJL mice, which is consistent with the differential severity of the disease at 35 dpi (Figure 3A). In the anti-KC antibody-treated SJL mice, both elevated demyelination and infiltration were found in all ventral, lateral, and dorsal areas of the spinal cord and the cellular infiltration was prominent in the demyelinated regions. In contrast, demyelination and infiltration in the control SJL mice were mild and mainly located in the ventral and lateral, but not dorsal spinal cord.

### Lower cellular infiltration into the CNS of anti-KC antibody-treated SJL mice compared to the control mice treated with normal IgG

**Figure 5:**
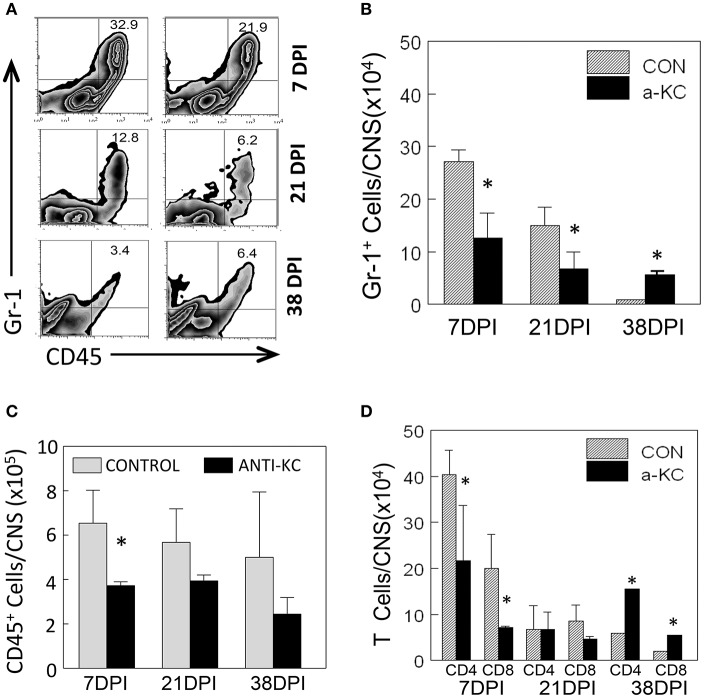
Cellular infiltration into the CNS of mice treated with anti-KC antibody and control mice. **(A)** The proportions of Gr-1^+^CD45^+^ neutrophils in the CNS of TMEV-infected mice treated with anti-KC and control antibodies were assessed using flow cytometry. **(B)** The numbers of Gr-1^+^CD45^+^ cells in mice treated with anti-KC antibody compared to control mice at early infection (7 dpi), during disease onset (21 dpi), and after the development of disease were assessed. The overall numbers of CD45^+^ cells **(C)** and CD4^+^ and CD8^+^ T cells **(D)** in the antibody-treated mice during the period of viral infection are shown. Values given are the means (± *SD*) of the results from 2 to 3 independent experiments. ^*^p < 0.05.

Because KC is known to affect the migration of neutrophils and other inflammatory cells (Zhou et al., 2003), we assessed the migration levels of Gr-1^+^CD45^+^ cells to the CNS of TMEV-infected mice treated with anti-KC or control antibody using flow cytometry (Figure 5). Gr-1^+^CD45^+^ cells include heterogeneous cell population with a myeloid origin (monocytes, neutrophils, and dendritic cells) associated with acute and chronic inflammations (Mazzoni et al., 2002; Soehnlein and Lindbom, 2010). The proportion and the number of Gr-1^+^CD45^+^ cells were significantly lower (*p* < 0.05) in mice treated with anti-KC antibody compared to the corresponding control mice at early infection (7 dpi) and during the onset of disease (21 dpi), but higher (*p* < 0.05) after the development of disease at 38 dpi (Figures 5A,B). Furthermore, the overall numbers of CD45^+^ cells and CD4^+^ and CD8^+^ T cells were significantly lower in the antibody-treated mice at the early (7 dpi) infection (Figures 5C,D). These results suggest that the reduced level of KC in the anti-KC antibody-treated mice significantly lowers the initial migration of neutrophils and other inflammatory cells including CD4^+^ and CD8^+^ T cells.

### Reduced levels of IFN-γ-producing CD4^+^ and CD8^+^ t cells in the CNS and periphery of anti-KC antibody-treated mice

**Figure 6:**
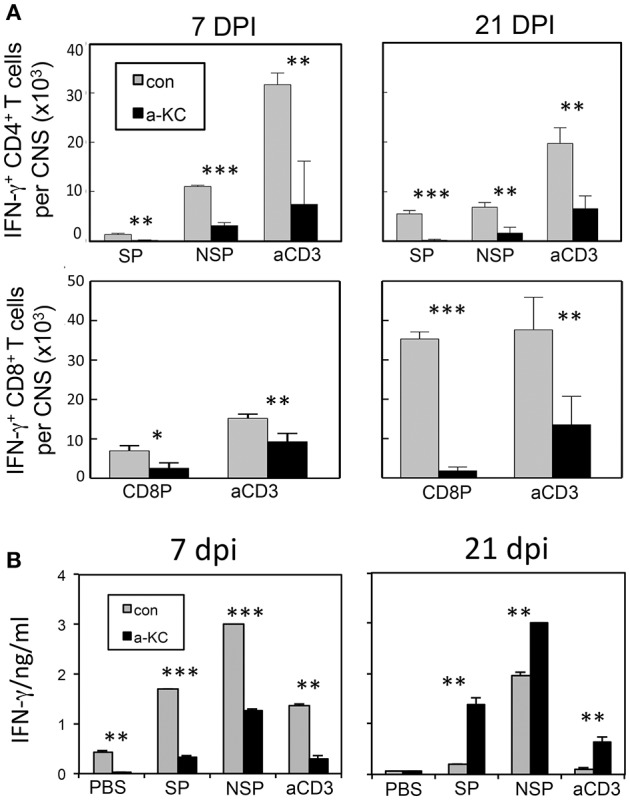
Analysis of CD4^+^ and CD8^+^ T cells that produce IFN-γ following infection with TMEV. **(A)** Mononuclear cells from the CNS of mice at 7 and 21 dpi were cultured for 6 h in the presence of PBS or a mixture of 1 μM of viral peptides and assessed the production of intracellular IFN-γ using flow cytometry. The data are representative of three independent experiments. **(B)** Splenocytes from mice at 7 and 21 dpi were cultured for 72 h in the presence of PBS, a mixture of 1 μM viral peptides or anti-CD3/CD28 antibodies. The production of IFN-γ in the culture supernatants were assessed using ELISA. Mixtures of CD4^+^ T cell-specific epitopes (SP mix; VP1_233–250_, VP2_74–86_, and VP3_24–37_; NSP mix, 3D_6–23_ and 3D_20–38_) and CD8 ^+^ T cell-specific epitopes (CD8 mix; VP3_159–166_, VP3_173–181_, and VP1_11–20_) were used. Mixtures of plate-bound anti-CD3 and anti-CD28 antibodies (1 μg each) were used to stimulate pan-T cells. Values given are the means (± *SD*) of the results from triplicate wells. ^*^p < 0.05; ^**^p < 0.01; ^***^p < 0.001.

To compare the levels of T cell responses to the viral determinants in mice treated with anti-KC antibodies and control IgGs, we assessed the levels of CD4^+^ and CD8^+^ T cells producing IFN-γ in the CNS following stimulation with viral peptides or anti-CD3/anti-CD28 pan-T cell stimulants (Figure 6). The results indicated that the numbers of IFN-γ-producing CD4^+^ T cells in the CNS prior to or at the onset of clinical symptoms (7 and 21 dpi) were significantly lower in mice treated with anti-KC antibodies (Figure 6A). The overall numbers of IFN-γ-producing CD8^+^ T cells in the CNS were also significantly lower in the anti-KC antibody-treated mice compared to the control (Figure 6A). These results strongly suggest that the treatment of TMEV-infected mice with anti-KC antibodies lowers the levels of protective IFN-γ-producing CD4^+^ and CD8^+^ T cells in the CNS. The levels of IFN-γ produced in the splenic cultures of mice treated with anti-KC antibodies at 7 dpi were similarly lower after stimulated with capsid and non-capsid epitope peptides (Figure 6B). Interestingly, however, the levels were higher at 21 dpi in contrast to the CNS. Elevated viral loads in the CNS of anti-KC treated mice due to the inferior protective T cell responses may result in the higher level of peripheral T cell response to the virus compared to the control mice.

### Production of functional KC upon infection of BHK cells with KC-expressing recombinant TMEV

To determine the potential role of KC in the pathogenesis of TMEV, we generated recombinant KC-TMEV based on the dL23 TMEV backbone. It was previously shown that recombinant TMEV derived from the dL23 TMEV construct retains its viral pathogenicity in highly susceptible SJL mice (Calenoff et al., 1995). Although there are stability problems associated with large inserts of foreign genes into TMEV genome (Zhang et al., 1995), the recombinant TMEVs expressing the small chemokine (KC) and control (HEL; hen egg lysozyme) genes were very stable. Additionally, no significant deletion of the foreign genes was found during later passages (>10) of the BHK cell line.

**Figure 7:**
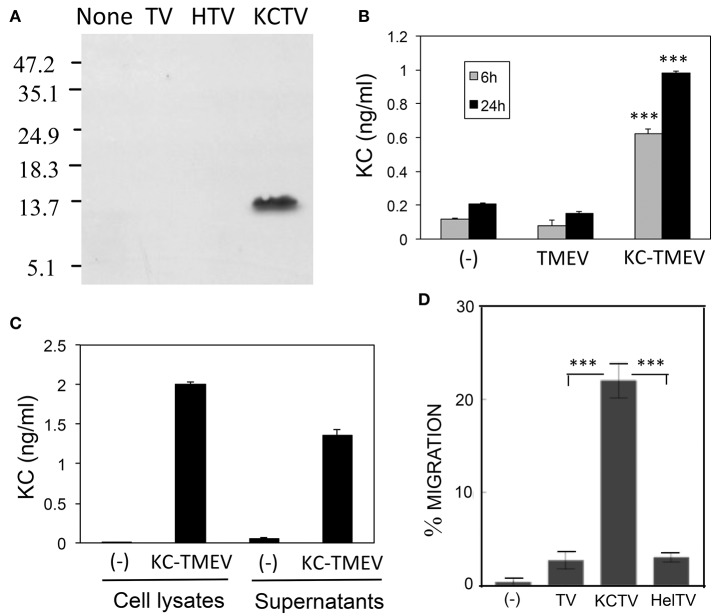
**(A)** Production of CXCL-1 (KC) following infection (MOI = 10) in BHK cells with control TMEV (TV), control HEL-TMEV (HTV), and KC-TMEV (KCTV) analyzed 24-h post infection with Western blotting. Cell lysates in Tris-buffered saline with 1% Triton X-100 were separated by electrophoresis, transferred to nitrocellulose membranes, and then analyzed using rabbit anti-KC antibody. A representative result from two similar experiments is shown. **(B)** BHK cell lysates from triplicate cultures that were mock-infected, or infected with control TMEV or KC-TMEV for 6 and 24 h were subjected to a mouse KC-specific ELISA (BD, CA). The means (± *SD*) of the results from triplicate cultures are shown. **(C)** To further determine whether the KC produced after infection with KC-TMEV remained cell bound or was secreted, culture supernatants and cell lysates of BHK cells mock-infected or infected with KC-TMEV were assessed for the presence of mouse KC using ELISA. The means (± *SD*) of the results from triplicate cultures are shown. **(D)** Supernatants of monolayer BHK cells mock-infected or infected with control TMEV or KC-TMEV for 24 h were overlaid with Boyden chamber membranes containing GFP-expressing peripheral blood neutrophils that were enriched by using negative magnetic sorting. The cultures were incubated for 3 h and the membranes were flipped over to assess the number of transmigrated cells. An example microscopy image of an experiment is shown. A summary of three transmigration assessments is shown. Values given are the means (± *SD*) of the results from three separate experiments. ^***^p < 0.001.

First, we assessed the production of KC in BHK cultures infected with KC-TMEV at 24 h post-infection using Western blotting against rabbit polyclonal antibodies to mouse KC (Figure 7A). Only the BHK cells infected with KC-TMEV showed detectable levels of mouse KC but not the cells infected with control TMEV or HEL-TMEV. The mouse KC-specific ELISA also indicated that BHK cells mock-infected or infected with control TMEV did not produced significant levels of KC at both 6 and 24 h post-infection (Figure 7B). In contrast, the levels of KC were several folds higher (*p* < 0.01) in BHK cells infected with KC-TMEV after 6 and 24 h. These results indicated that the cells infected with KC-TMEV produce higher level of KC than the controls. Further experiments showed that KC was present in culture supernatants as well as in the cell lysates (Figure 7C). Therefore, the high levels of KC produced following KC-TMEV infection might affect the function of both infected cells and adjacent cells.

To further determine the functionality of KC molecules produced after infection with KC-TMEV, the degree of cell migration in the presence or absence of the chemokine were examined using cell transmigration assays as previously described (Gho et al., 1999). The transmigration of peripheral blood neutrophils that were purified using negative selection was tested to detect whether the cells could migrate through micropore membranes via the KC gradient produced after KC-TMEV infection (Figure 7D). The peripheral neutrophils were placed on top of Boyden chamber membranes and incubated for 3 h with serum-free culture supernatants from BHK cells mock-infected, or infected with TMEV, HEL-TMEV, or KC-TMEV for 24 h. The results clearly indicated that the transmigration of neutrophils into the side of the chamber containing supernatants of BHK cells infected with KC-TMEV was significantly increased (*p* < 0.001) compared to that of mock-infected or control HEL-TMEV or TMEV-infected cells. Therefore, these results strongly suggest that the KC molecules produced following infection with recombinant KC-TMEV are biologically active in promoting cellular migration.

### Elevated pathogenesis of demyelinating disease after KC-TMEV infection

**Figure 8:**
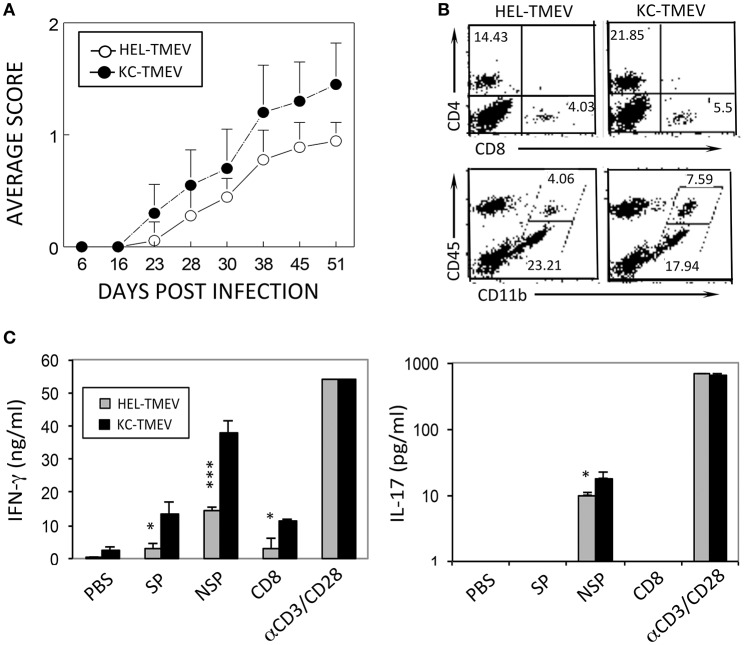
Development of demyelinating disease and T cell responses after infection with KC-TMEV and control HEL-TMEV. **(A)** SJL mice were infected with KC-TMEV (*n* = 10) or control HEL-TMEV (*n* = 9). The animals were graded for clinical signs as described in the Materials and Methods section. The results are expressed as average clinical scores of animals at the indicated dpi. The two-tailed *p*-value of the two groups based on the paired *t*-test between 23 and 51 dpi was very significant (*p* < 0.0004). **(B)** Infiltration of CD4^+^ and CD8^+^ T cells and CD45^+^ CD11b^+^ cells into the CNS after infection with KC-TMEV or TMEV (4 dpi) was assessed using flow cytometry. The overall numbers of CD4^+^ T cells [(43.421 ± 6.585 vs. 11.339 ± 2.849) × 10^4^] and macrophages [(10.627 ± 3.474 vs. 2.342 ± 1.426) × 10^4^] infiltrated in the CNS of KC-TMEV infected mice were significantly higher than those of HEL-TMEV infected mice, respectively. **(C)** IFN-γ and IL-17 levels in the supernatants of the splenic cultures were determined using ELISA. Spleen cells were prepared 8 dpi from SJL mice infected with control HEL-TMEV (*n* = 3) or KC-TMEV (*n* = 3). Cells were cultured for 72 h in the presence of PBS or a mixture of 1 μM of the peptides. Mixtures of CD4^+^ T-cell-specific epitopes (SP mix; VP1_233–250_, VP2_74–86_, and VP3_24–37_; NSP mix, 3D_6–23_ and 3D_20–38_) and CD8^+^ T cell-specific epitopes (CD8 mix; VP3_159–166_, VP3_173–181_, and VP1_11–20_) were used. αCD3/CD28 represents plate-bound anti-CD3 and anti-CD28 antibodies (1 μg each) used to stimulate pan-T cells. The culture supernatants were subsequently collected for ELISA. The data are representative of three independent experiments. Values given are the means (± *SD*) of the results from triplicate wells. ^*^p < 0.05; ^***^p < 0.001.

To further determine the role of KC produced during KC-TMEV infection in the pathogenesis of demyelinating disease, we compared the course of the disease development following infection of SJL mice (*n* = 10 and *n* = 9, respectively) with KC-TMEV and control HEL-TMEV (Figure 8A). The development of demyelinating disease after infection with KC-TMEV was significantly accelerated (*p* < 0.001) during the course of 50 days compared to mice infected with HEL-TMEV. Therefore, the presence of an elevated level of KC during viral infection appears to play an important pathogenic role in the development of demyelinating disease.

We further compared the levels of CD4^+^ and CD8^+^ T cells, and CD45^+^ cells in conjunction with the expression of CD11b in the CNS of mice infected with KC-TMEV and HEL-TMEV (Figure 8B). The results indicated that CD4^+^ T cells and CD11b^+^CD45^+^ macrophages, in particular, infiltrated into the CNS at higher levels in the KC-TMEV infected mice. In combination with nearly 2-fold higher infiltration of overall mononuclear cells and higher proportions in the CNS (Figure 8B), the overall numbers of macrophages (*p* = 0.0187) and CD4^+^ T cells (*p* = 0.0015) were 3 to 4-fold higher in the CNS of KC-TMEV infected mice compared to the control HEL-TMEV infected mice. We also examined IFN–γ and IL-17 production in response to the CD4^+^ and CD8^+^ T cell-specific viral epitopes in these infected mice at 8 dpi (Figure 8C). The level of IFN-γ production in KC-TMEV infected mice was significantly higher (*p* < 0.05–0.001) than those in the control HEL-TMEV infected mice. In addition, the level of IL-17 production was increased (*p* < 0.05) in the KC-TMEV infected mice, indicating that these mice, which showed elevated pathogenesis of demyelination, also display elevated pathogenic inflammatory T cell responses. Therefore, although an excessive level of KC may promote the pathogenesis of the disease, but the lack of KC can also result in disease pathogenesis because of the failure to recruit cells that are required to protect against viral infection.

## Discussion

TMEV infection into various cell types, including different glial cells and antigen-presenting cells, similar to other viral infections, rapidly induces the production of several dominant chemokines (Palma and Kim, 2001, 2004). KC is one of the most predominant chemokines produced in various glial cell types within hours after TMEV infection (Palma and Kim, 2004; Rubio and Sanz-Rodriguez, 2007). Previously, the effects of some of these chemokines such as CCL5, CCL9 (Ure et al., 2005), and CCL2 (Karpus et al., 2006) on the development of TMEV-induced demyelinating disease were reported. These studies indicated that treatment of mice with anti-chemokine antibodies increased viral loads and promoted the pathogenesis of the disease development. Similarly, other neurotropic virus-induced diseases were also investigated and found that these chemokines play a protective role (Hosking et al., 2009). However, very little is known about the role of KC, which is associated with the migration of first defense cell type neutrophils (Zhou et al., 2003) in the pathogenesis of TMEV-induced demyelinating disease. In this study, we have explored the potential role of one of the most abundantly produced chemokine, KC, in the development of TMEV-induced demyelinating disease by utilizing antibodies against KC and recombinant KC-TMEV carrying the KC gene.

It was interesting to observe that several-fold higher levels of KC were produced in cells from susceptible SJL mice compared to those from resistant B6 mice following TMEV infection and stimulations with different TLR ligands (Figure 1). These results are consistent with the previous finding that astrocytes derived from susceptible SJL mice but not astrocytes derived from resistant BALB/c mice, which are also resistant to TMEV-induced demyelinating disease, produce high levels of KC following TMEV infection (Rubio and Sanz-Rodriguez, 2007). These findings suggested that excessive production of KC following viral infection might contribute to the susceptibility of SJL mice to the development of TMEV-induced demyelinating disease, as seen from the effects of IL-1β, IFN-α/β, or TLR3 signaling (Jin et al., 2010, 2011; Kim et al., 2012). The higher levels of KC production upon TMEV infection may reflect the higher levels of viral permissiveness of the cells from susceptible SJL mice compared to the cells from resistant C57BL/6 mice (Jin et al., 2007, 2015). Interestingly, the production of KC depended on the presence of MyD88 (Figure 2), which is not associated with the major TMEV-induced TLR3 signaling (So et al., 2006; Johnson et al., 2008). Therefore, a secondary TLR or a cytokine signaling following TMEV infection may be involved in the triggering the KC production. It is conceivable that IL-1 and/or IL-18 produced in a MyD88-dependent manner may be associated with the production of KC as shown previously (Adachi et al., 1998; Lee et al., 2015). Because TMEV infection is known to induce the upregulation of other TLRs including TLR2 (Turrin, 2008; So and Kim, 2009) whose signaling depends on the presence of MyD88 (Takeda and Akira, 2004), it is likely that TLR2 signaling upregulated via the TMEV-induced TLR3 activation is associated with KC production.

Treatment of mice with anti-KC antibodies significantly promoted the pathogenesis of TMEV-induced demyelinating disease and elevated viral loads in the CNS compared to control IgG-treated mice (Figure 3). Therefore, the presence of KC during the initial viral infection is essential for the protection from the development of TMEV-induced demyelinating disease. Direct assessment of KC gradient differences in the CNS during viral infections, although technically challenging, may be helpful in understanding the role of KC in the pathogenesis of demyelinating disease in future studies. The development of clinical signs and high viral loads in the CNS were consistent with histopathological examinations of the spinal cords, which showed increased irregular vacuolation and demyelinating lesions in the white matter of the spinal cords of anti-KC antibody-treated mice compared to the control mice (Figure 4). The TMEV BeAn strain, unlike DA strain, is known to induce minimal to undetectable acute phase disease (Dal Canto and Lipton, 1975; Dal Canto et al., 1996). SJL mice infected with BeAn strain induces a progressive demyelinating disease as early as 12–20 days post-infection depending on the potency of the viral batch and their immune status (Hou et al., 2007, 2009). Therefore, it is most likely that our observed clinical symptoms represent the progression of demyelinating disease, coupled with the histological evidence at 35 dpi (Figure 4). In addition, the infiltration of Gr-1^+^CD45^+^ cells and the overall numbers of CD45^+^ cells, including CD4^+^ and CD8^+^ T cells, into the CNS was significantly lower in mice treated with anti-KC antibody compared to the control mice at early infection and during disease onset (Figure 5). Consistent with the infiltration of T cell levels, the protective IFN-γ-producing T cell levels in the CNS were also lower in the antibody-treated mice (Figure 6). These results strongly suggest that a low level of KC during viral infection fails to facilitate the migration of sufficient neutrophils and other inflammatory cells into the CNS at the site of viral infection and propagation. These results are consistent with the previous findings with mouse hepatitis virus-induced demyelinating disease following the treatment with anti-CCR2 antibody against the CXCL1 receptor (Hosking et al., 2009). Therefore, the presence of KC at the site of initial viral infection appears to play an important role in the protection against viral pathogenesis by promoting cellular infiltration into the CNS.

To further determine the effects of excessive KC production on the pathogenesis of TMEV, we generated recombinant KC-TMEV based on the dL23 TMEV backbone (Calenoff et al., 1995). Western blotting with anti-mouse KC antibodies and mouse KC-specific ELISA on virus-infected BHK cells indicated that the levels of KC produced in the KC-TMEV infected cells for 6 and 24 h were several folds higher than that in the cells infected with control HEL-TMEV (Figure 7). The transmigration of purified peripheral blood neutrophils through micropore membranes due to KC production after KC-TMEV infection was also significantly higher in the KC-TMEV infected cells, indicating the intact functionality of KC produced by the recombinant KC-TMEV. Therefore, excessive production of KC following KC-TMEV infection is most likely to affect the migration and/or activation of both infected cells and the adjacent cells.

The development of demyelinating disease in SJL mice infected with KC-TMEV was significantly accelerated compared to mice infected with control HEL-TMEV (Figure 8). Thus, the presence of an excessive level of KC during KC-TMEV infection appears to play a pathogenic role in the development of demyelinating disease. Neutrophils, T cells, and macrophages infiltrated the CNS at higher levels in the KC-TMEV-infected mice, which showed elevated pathogenesis of demyelination. Interestingly, the KC-TMEV infected mice also displayed elevated inflammatory T cell responses, which are known to be responsible for the pathogenesis of demyelination (Myoung et al., 2008). This result is opposite to the effect of KC on the development of autoimmune demyelinating disease in inducible KC-Tg mice (Omari et al., 2009), which provided protection from the disease development. However, infection of the Tg mice displaying sustained KC expression with mouse hepatitis virus resulted in increased mortality accompanying elevated neutrophil infiltration induced demyelinating disease (Marro et al., 2016). These results indicates that the role of KC in the pathogenesis of TMEV-induced demyelinating disease may be dual similar to other virus-induced CNS inflammatory diseases, i.e., its presence is helpful for protection but excessive levels can be pathogenic, similar to the roles of IL-1. We have previously shown that the presence of a high level of IL-1 plays a pathogenic role, whereas the lack of IL-1 signal promotes viral persistence in the spinal cord due to insufficient T cell activation (Kim et al., 2012). In a similar way, an excessive level of TLR3 signaling contributes to disease pathogenesis, but the presence of the TLR3 signal is required to protect the host from chronic viral infections (Jin et al., 2011).

## Conclusions

In this study, we first compared the levels of KC, one of the most predominant chemokines upon viral infection, produced after infection with TMEV or stimulation with various TLRs in various cells from susceptible SJL mice and resistant B6 mice. The level of KC produced after infection with TMEV or stimulation with TLRs was significantly higher in cells from susceptible SJL mice than those in cells from resistant B6 mice. In addition, the production of KC was completely dependent on the presence of MyD88, unlike the production of IL-6. This result strongly suggests that the pathways associated with the production of KC is distinct from that of IL-6, which is one of the most important cytokine affecting the pathogenesis of viral demyelinating disease (Rodriguez et al., 1994; Hou et al., 2009, 2014; Jin et al., 2013). We further investigated the potential role of KC in the progression of TMEV-induced demyelinating disease. We have either reduced the level of KC with anti-KC antibody treatment or supplied an excessive level of KC during viral infection with recombinant KC-TMEV producing KC. Our results indicated that the presence of a certain level of this chemokine is essential for the influx of cells into the CNS for protection, but an excessive amount can be harmful because of the induction of inflammation during virally induced chronic demyelinating disease. Our approach of utilizing the recombinant virus expressing the chemokine is particularly important because it allows the chemokine to be targeted at the infection site at a level proportional to viral replication without any further manipulation. A variety of biologically active small molecules, such as IL-1, may also be applied in the construction of recombinant viruses to investigate the function and role of these molecules in the pathogenesis of chronic virally induced inflammatory diseases.

## Ethics statement

Experiments using animals were conducted according to the permission (#2011-1316 for Byung Kim) of the Animal Care and Use Committee at Northwestern University.

## Author contributions

MK carried out the initial experiments determining the expression of KC and IL-6 genes in cells infected with TMEV or stimulated with TLR ligands. Also, MK generated KC-TMEV and HEL-TMEV recombinant viruses and characterized the viruses. MK and YJ conducted the animal experiments including the disease development and various immune assays. BK directed the experiments and prepared the manuscript with YJ.

### Conflict of interest statement

The authors declare that the research was conducted in the absence of any commercial or financial relationships that could be construed as a potential conflict of interest.

## Abbreviations

CNS: central nervous system
KC: keratinocyte-derived cytokine
dpi: days post-infection
CNS: central nervous system
TMEV: Theiler's murine encephalomyelitis virus
TMEV-IDD: TMEV-induced demyelinating disease.
KC-TMEV: KC-expressing recombinant TMEV
HEL-TMEV: hen-egg lysozyme-expressing TMEV
PCR: polymerase chain reaction
PFU: plaque-forming unit
MOI: multiplicity of infection.

## References

[B1] Adachi O. Kawai T. Takeda K. Matsumoto M. Tsutsui H. Sakagami M.. (1998). Targeted disruption of the MyD88 gene results in loss of IL-1- and IL-18-mediated function. Immunity 9, 143–150. 10.1016/S1074-7613(00)80596-8 9697844

[B2] Aubert C. Chamorro M. Brahic M. (1987). Identification of Theiler's virus infected cells in the central nervous system of the mouse during demyelinating disease. Microb. Pathog. 3, 319–326. 10.1016/0882-4010(87)90002-7 2849023

[B3] Calenoff M. A. Badshah C. S. Dal Canto M. C. Lipton H. L. Rundell M. K. (1995). The leader polypeptide of Theiler's virus is essential for neurovirulence but not for virus growth in BHK cells. J. Virol. 69, 5544–5549. 7636999 10.1128/jvi.69.9.5544-5549.1995 PMC189406

[B4] Clatch R. J. Lipton H. L. Miller S. D. (1987). Class II-restricted T cell responses in *Theiler's murine* encephalomyelitis virus (TMEV)-induced demyelinating disease. II. Survey of host immune responses and central nervous system virus titers in inbred mouse strains. Microb. Pathog. 3, 327–337. 10.1016/0882-4010(87)90003-9 2849024

[B5] Cotter M. J. Norman K. E. Hellewell P. G. Ridger V. C. (2001). A novel method for isolation of neutrophils from murine blood using negative immunomagnetic separation. Am. J. Pathol. 159, 473–481. 10.1016/S0002-9440(10)61719-1 11485906 PMC1850545

[B6] Dal Canto M. C. Lipton H. L. (1975). Primary demyelination in Theiler's virus infection. an ultrastructural study. Lab. Invest. 33, 626–637. 1202282

[B7] Dal Canto M. C. Kim B. S. Miller S. D. Melvold R. W. (1996). Theiler's Murine encephalomyelitis virus (TMEV)-induced demyelination: a model for human multiple sclerosis. Methods 10, 453–461. 10.1006/meth.1996.0123 8954856

[B8] Farnand A. W. Eastman A. J. Herrero R. Hanson J. F. Mongovin S. Altemeier W. A.. (2011). Fas activation in alveolar epithelial cells induces KC (CXCL1) release by a MyD88-dependent mechanism. Am. J. Respir. Cell Mol. Biol. 45, 650–658. 10.1165/rcmb.2010-0153OC 21257927 PMC3175574

[B9] Gho Y. S. Kleinman H. K. Sosne G. (1999). Angiogenic activity of human soluble intercellular adhesion molecule-1. Cancer Res. 59, 5128–5132. 10537287

[B10] Guerrero A. T. Cunha T. M. Verri W. A. Jr. Gazzinelli R. T. Teixeira M. M. Cunha F. Q.. (2012). Toll-like receptor 2/MyD88 signaling mediates zymosan-induced joint hypernociception in mice: participation of TNF-alpha, IL-1beta and CXCL1/KC. Eur. J. Pharmacol. 674, 51–57. 10.1016/j.ejphar.2011.10.023 22051147

[B11] Hosking M. P. Liu L. Ransohoff R. M. Lane T. E. (2009). A protective role for ELR+ chemokines during acute viral encephalomyelitis. PLoS Pathog. 5:e1000648. 10.1371/journal.ppat.1000648 19893623 PMC2766051

[B12] Hou W. Jin Y. H. Kang H. S. Kim B. S. (2014). Interleukin-6 (IL-6) and IL-17 synergistically promote viral persistence by inhibiting cellular apoptosis and cytotoxic T cell function. J. Virol. 88, 8479–8489. 10.1128/JVI.00724-14 24829345 PMC4135960

[B13] Hou W. Kang H. S. Kim B. S. (2009). Th17 cells enhance viral persistence and inhibit T cell cytotoxicity in a model of chronic virus infection. J. Exp. Med. 206, 313–328. 10.1084/jem.20082030 19204109 PMC2646583

[B14] Hou W. So E. Y. Kim B. S. (2007). Role of dendritic cells in differential susceptibility to viral demyelinating disease. PLoS Pathog. 3:e124. 10.1371/journal.ppat.0030124 17722981 PMC1950949

[B15] Jin Y. H. Hou W. Kang H. S. Koh C. S. Kim B. S. (2013). The role of interleukin-6 in the expression of PD-1 and PDL-1 on central nervous system cells following infection with Theiler's murine encephalomyelitis virus. J. Virol. 87, 11538–11551. 10.1128/JVI.01967-13 23966393 PMC3807328

[B16] Jin Y. H. Hou W. Kim S. J. Fuller A. C. Kang B. Goings G.. (2010). Type I interferon signals control Theiler's virus infection site, cellular infiltration and T cell stimulation in the CNS. J. Neuroimmunol. 226, 27–37. 10.1016/j.jneuroim.2010.05.028 20538350 PMC2937062

[B17] Jin Y. H. Kaneyama T. Kang M. H. Kang H. S. Koh C. S. Kim B. S. (2011). TLR3 signaling is either protective or pathogenic for the development of Theiler's virus-induced demyelinating disease depending on the time of viral infection. J. Neuroinflammation 8:178. 10.1186/1742-2094-8-178 22189096 PMC3293102

[B18] Jin Y. H. Kang H. S. Hou W. Meng L. Kim B. S. (2015). The level of viral infection of antigen-presenting cells correlates with the level of development of Theiler's murine encephalomyelitis virus-induced demyelinating disease. J. Virol. 89, 1867–1878. 10.1128/JVI.02471-14 25428872 PMC4300758

[B19] Jin Y. H. Kim S. J. So E. Y. Meng L. Colonna M. Kim B. S. (2012). Melanoma differentiation-associated gene 5 is critical for protection against Theiler's virus-induced demyelinating disease. J. Virol. 86, 1531–1543. 10.1128/JVI.06457-11 22090123 PMC3264388

[B20] Jin Y. H. Mohindru M. Kang M. H. Fuller A. C. Kang B. Gallo D.. (2007). Differential virus replication, cytokine production, and antigen-presenting function by microglia from susceptible and resistant mice infected with Theiler's virus. J. Virol. 81, 11690–11702. 10.1128/JVI.01034-07 17715222 PMC2168808

[B21] Johnson A. C. Li X. Pearlman E. (2008). MyD88 functions as a negative regulator of TLR3/TRIF-induced corneal inflammation by inhibiting activation of c-Jun N-terminal kinase. J. Biol. Chem. 283, 3988–3996. 10.1074/jbc.M707264200 18057004

[B22] Karpus W. J. Kennedy K. J. Fife B. T. Bennett J. L. Dal Canto M. C. Kunkel S. L.. (2006). Anti-CCL2 treatment inhibits Theiler's murine encephalomyelitis virus-induced demyelinating disease. J. Neurovirol. 12, 251–261. 10.1080/13550280600873819 16966216 PMC4040265

[B23] Kerstetter A. E. Padovani-Claudio D. A. Bai L. Miller R. H. (2009). Inhibition of CXCR2 signaling promotes recovery in models of multiple sclerosis. Exp. Neurol. 220, 44–56. 10.1016/j.expneurol.2009.07.010 19616545 PMC2761527

[B24] Kim B. S. Jin Y. H. Meng L. Hou W. Kang H. S. Park H. S.. (2012). IL-1 signal affects both protection and pathogenesis of virus-induced chronic CNS demyelinating disease. J. Neuroinflammation 9:217. 10.1186/1742-2094-9-217 22985464 PMC3462702

[B25] Kim B. S. Lyman M. A. Kang B. S. Kang H. K. Lee H. G. Mohindru M.. (2001). Pathogenesis of virus-induced immune-mediated demyelination. Immunol. Res. 24, 121–130. 10.1385/IR:24:2:121 11594451 PMC7091353

[B26] Lee Y. S. Yang H. Yang J. Y. Kim Y. Lee S. H. Kim J. H. (2015). Interleukin-1 (IL-1) signaling in intestinal stromal cells controls KC/ CXCL1 secretion, which correlates with recruitment of IL-22- secreting neutrophils at early stages of Citrobacter rodentium infection. Infect. Immun. 83, 3257–3267. 10.1128/IAI.00670-15 26034212 PMC4496604

[B27] Lehrich J. R. Arnason B. G. Hochberg F. H. (1976). Demyelinative myelopathy in mice induced by the DA virus. J. Neurol. Sci. 29, 149–160. 10.1016/0022-510X(76)90167-2 185333

[B28] Leibowitz J. L. Rodriguez M. (1983). Antigenic variants are not selected during persistent infection with Theiler's virus. Infect. Immun. 41, 440–442. 6190757 10.1128/iai.41.1.440-442.1983 PMC264801

[B29] Lipton H. L. Dal Canto M. C. (1976). Theiler's virus-induced demyelination: prevention by immunosuppression. Science 192, 62–64. 10.1126/science.176726 176726

[B30] Lipton H. L. Kumar A. S. Trottier M. (2005). Theiler's virus persistence in the central nervous system of mice is associated with continuous viral replication and a difference in outcome of infection of infiltrating macrophages versus oligodendrocytes. Virus Res. 111, 214–223. 10.1016/j.virusres.2005.04.010 15893838

[B31] Lorch Y. Friedmann A. Lipton H. L. Kotler M. (1981). Theiler's murine encephalomyelitis virus group includes two distinct genetic subgroups that differ pathologically and biologically. J. Virol. 40, 560–567. 6275104 10.1128/jvi.40.2.560-567.1981 PMC256659

[B32] Marro B. S. Grist J. J. Lane T. E. (2016). Inducible expression of CXCL1 within the central nervous system amplifies viral-induced demyelination. J. Immunol. 196, 1855–1864. 10.4049/jimmunol.1501802 26773148 PMC4742654

[B33] Mazzoni A. Bronte V. Visintin A. Spitzer J. H. Apolloni E. Serafini P.. (2002). Myeloid suppressor lines inhibit T cell responses by an NO-dependent mechanism. J. Immunol. 168, 689–695. 10.4049/jimmunol.168.2.689 11777962

[B34] Myoung J. Bahk Y. Y. Kang H. S. Dal Canto M. C. Kim B. S. (2008). Anticapsid immunity level, not viral persistence level, correlates with the progression of Theiler's virus-induced demyelinating disease in viral P1-transgenic mice. J. Virol. 82, 5606–5617. 10.1128/JVI.02442-07 18353953 PMC2395227

[B35] Omari K. M. Lutz S. E. Santambrogio L. Lira S. A. Raine C. S. (2009). Neuroprotection and remyelination after autoimmune demyelination in mice that inducibly overexpress CXCL1. Am. J. Pathol. 174, 164–176. 10.2353/ajpath.2009.080350 19095949 PMC2631329

[B36] Palma J. P. Kim B. S. (2001). Induction of selected chemokines in glial cells infected with Theiler's virus. J. Neuroimmunol. 117, 166–170. 10.1016/S0165-5728(01)00326-5 11431017

[B37] Palma J. P. Kim B. S. (2004). The scope and activation mechanisms of chemokine gene expression in primary astrocytes following infection with Theiler's virus. J. Neuroimmunol. 149, 121–129. 10.1016/j.jneuroim.2003.12.025 15020072

[B38] Pullen L. C. Park S. H. Miller S. D. Dal Canto M. C. Kim B. S. (1995). Treatment with bacterial LPS renders genetically resistant C57BL/6 mice susceptible to Theiler's virus-induced demyelinating disease. J. Immunol. 155, 4497–4503. 7594613

[B39] Robinson S. Tani M. Strieter R. M. Ransohoff R. M. Miller R. H. (1998). The chemokine growth-regulated oncogene-alpha promotes spinal cord oligodendrocyte precursor proliferation. J. Neurosci. 18, 10457–10463. 9852583 10.1523/JNEUROSCI.18-24-10457.1998 PMC6793357

[B40] Rodriguez M. Pavelko K. D. McKinney C. W. Leibowitz J. L. (1994). Recombinant human IL-6 suppresses demyelination in a viral model of multiple sclerosis. J. Immunol. 153, 3811–3821. 7930598

[B41] Rubio N. Sanz-Rodriguez F. (2007). Induction of the CXCL1 (KC) chemokine in mouse astrocytes by infection with the murine encephalomyelitis virus of Theiler. Virology 358, 98–108. 10.1016/j.virol.2006.08.003 16996102

[B42] Skias D. D. Kim D. K. Reder A. T. Antel J. P. Lancki D. W. Fitch F. W. (1987). Susceptibility of astrocytes to class I MHC antigen-specific cytotoxicity. J. Immunol. 138, 3254–3258. 3106479

[B43] So E. Y. Kim B. S. (2009). Theiler's virus infection induces TLR3-dependent upregulation of TLR2 critical for proinflammatory cytokine production. Glia 57, 1216–1226. 10.1002/glia.20843 19191335 PMC2706926

[B44] So E. Y. Kang M. H. Kim B. S. (2006). Induction of chemokine and cytokine genes in astrocytes following infection with Theiler's murine encephalomyelitis virus is mediated by the Toll-like receptor 3. Glia 53, 858–867. 10.1002/glia.20346 16586493

[B45] Soehnlein O. Lindbom L. (2010). Phagocyte partnership during the onset and resolution of inflammation. Nat. Rev. Immunol. 10, 427–439. 10.1038/nri2779 20498669

[B46] Takeda K. Akira S. (2004). TLR signaling pathways. Semin. Immunol. 16, 3–9. 10.1016/j.smim.2003.10.003 14751757

[B47] Tsai H. H. Frost E. To V. Robinson S. Ffrench-Constant C. Geertman R.. (2002). The chemokine receptor CXCR2 controls positioning of oligodendrocyte precursors in developing spinal cord by arresting their migration. Cell 110, 373–383. 10.1016/S0092-8674(02)00838-3 12176324

[B48] Turrin N. P. (2008). Central nervous system Toll-like receptor expression in response to Theiler's murine encephalomyelitis virus-induced demyelination disease in resistant and susceptible mouse strains. Virol. J. 5:154. 10.1186/1743-422X-5-154 19094215 PMC2614974

[B49] Ure D. R. Lane T. E. Liu M. T. Rodriguez M. (2005). Neutralization of chemokines RANTES and MIG increases virus antigen expression and spinal cord pathology during Theiler's virus infection. Int. Immunol. 17, 569–579. 10.1093/intimm/dxh236 15824069 PMC7108597

[B50] Yauch R. L. Palma J. P. Yahikozawa H. Koh C. S. Kim B. S. (1998). Role of individual T-cell epitopes of Theiler's virus in the pathogenesis of demyelination correlates with the ability to induce a Th1 response. J. Virol. 72, 6169–6174. 9621084 10.1128/jvi.72.7.6169-6174.1998 PMC110426

[B51] Yona S. Hayhoe R. Avraham-Davidi I. (2010). Monocyte and neutrophil isolation and migration assays. Curr. Protoc. Immunol. Chapter 14:Unit 14.15. 10.1002/0471142735.im1415s88 20143313

[B52] Zhang L. Sato S. Kim J. I. Roos R. P. (1995). Theiler's virus as a vector for foreign gene delivery. J. Virol. 69, 3171–3175. 7707546 10.1128/jvi.69.5.3171-3175.1995 PMC189020

[B53] Zheng L. Calenoff M. A. Dal Canto M. C. (2001). Astrocytes, not microglia, are the main cells responsible for viral persistence in Theiler's murine encephalomyelitis virus infection leading to demyelination. J. Neuroimmunol. 118, 256–267. 10.1016/S0165-5728(01)00338-1 11498260

[B54] Zhou J. Stohlman S. A. Hinton D. R. Marten N. W. (2003). Neutrophils promote mononuclear cell infiltration during viral-induced encephalitis. J. Immunol. 170, 3331–3336. 10.4049/jimmunol.170.6.3331 12626593

